# The Regulatory Effects of mTOR Complexes in the Differentiation and Function of CD4^+^ T Cell Subsets

**DOI:** 10.1155/2020/3406032

**Published:** 2020-04-23

**Authors:** Peng Wang, Qian Zhang, Liang Tan, Yanan Xu, Xubiao Xie, Yong Zhao

**Affiliations:** ^1^State Key Laboratory of Membrane Biology, Institute of Zoology, Chinese Academy of Sciences; University of Chinese Academy of Sciences, Beijing, China; ^2^Department of Immunology, School of Basic Medical Sciences, Zhengzhou University, Zhengzhou, China; ^3^University of Chinese Academy of Sciences, Beijing, China; ^4^Department of Urological Organ Transplantation, Center of Organ Transplantation, The Second Xiangya Hospital of Central South University, Changsha, China; ^5^Institute for Stem Cell and Regeneration, Chinese Academy of Sciences, Beijing, China

## Abstract

T cells are an important part of the adaptive immune system and play critical roles in the elimination of various pathogens. T cells could differentiate into distinct cellular subsets under different extracellular signals and then play different roles in maintaining host homeostasis and defense. The mechanistic target of rapamycin (mTOR) is a conserved intracellular serine/threonine kinase which belongs to the phosphoinositide 3-kinase- (PI3K-) related kinase family. The mTOR signaling pathway is closely involved in a variety of cell biological processes, including cell growth and cell metabolism, by senses and integrates various environmental cues. Recent studies showed that mTOR including mTORC1 and mTORC2 is closely involved in the development of T cell subpopulations such as Th1, Th2, Th9, Th17, follicular helper T cells (Tfh), and Treg cells through distinctive pathways. We herein mainly focused on the recent progress in understanding the roles of mTOR in regulating the development and differentiation of CD4^+^ T cell subsets.

## 1. Introduction

T cells are the central element of the adaptive immune system for their functions in eliminating viral, bacterial, parasitic, or other pathogens and antigens. After maturation in the thymus, T cells enter and circulate in the blood and lymphatic systems and then reside in the lymph nodes and other secondary lymphoid organs. When organisms encounter foreign pathogens and antigens, naïve T cells will be activated by MHC antigenic peptides and costimulatory signals of antigen-presenting cells (APCs). These activated T cells will then perform effector functions through secreting various cytokines or cytotoxicity. In different local microenvironments, activated CD4^+^ T cells will differentiate into distinct T cells, which participate in various immune response or autoimmunity mainly by producing various cytokines. Cytotoxic CD8^+^ T cells directly kill infected cells or malignant cells. During the process of development and differentiation of T cells, lots of signaling pathways play critical roles to orchestrate the cell fate decision, cell survival, and cell functions.

In the 1990s, the target of rapamycin (TOR) was found as a mediator of the toxic effect of rapamycin in yeast [1, 2]. TOR was proved as the target of rapamycin, which is an antifungal macrolide produced by the bacterial species *Streptomyces hygroscopicus*. The mechanistic target of rapamycin (mTOR), also known as the mammalian target of rapamycin, is a conserved serine/threonine kinase, which is a member of the phosphatidylinositol 3-kinase- (PI3K-) related kinase (PI3KK) family, and plays an important role in the signaling network that controls growth and metabolism in response to environmental cues. The kinase mTOR plays a central regulatory role in lots of biological processes of an organism, including metabolism, protein synthesis, energy balance, proliferation, and survival [3]. Recently, it was found that mTOR also plays important roles in the immune system and that mTOR acts as a key molecule in sensing immune microenvironment and dictating functions and differentiation of immune cells [4]. In the present review, we will focus on the different roles of mTOR in mastering the differentiation of distinctive Th cell subsets.

## 2. Structure and Functions of mTOR

**Figure 1:**
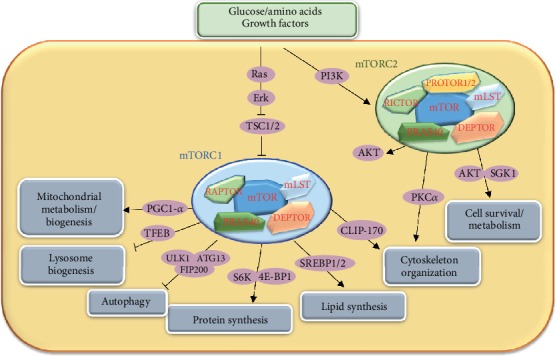
mTOR signaling pathway and its functions. After receiving the extracellular signals, including glucose, amino acids, or growth factor, a series of signaling molecules (Ras, PI3K, AKT, and so on) upstream of mTORC1 are activated; then, mTORC1 and its downstream molecules are activated to regulate a series of cellular processes, such as protein synthesis, autophagy, and mitochondrial metabolism. How mTORC2 is regulated has not been thoroughly studied. mTORC2 mainly regulates cell survival, metabolism, and cytoskeleton organization through SGK1 and PKC*α*, respectively. mTORC1 and mTORC2 are linked through AKT.

The mTOR complex at least includes two kinds of distinct multimolecular signaling forms, mTOR complex 1 (mTORC1) and mTOR complex 2 (mTORC2), both of which comprise a common molecule mTOR which is the catalytic subunit (Figure 1). It has been proved that deletion of mTOR resulted in the loss of activity of mTORC1 and mTORC2 [5]. Besides the basic molecule mTOR, mTORC1 comprises other four subunits: the scaffolding protein regulatory-associated protein of mTOR (RAPTOR), which might regulate the assembly of the complex and recruiting substrates for mTOR; DEP-containing mTOR-interacting protein (DEPTOR) and proline-rich AKT substrate 40 kDa (PRAS40), which has been proved to negatively regulate the activity of mTORC1; and mammalian lethal with Sec13 protein 8 (mLST8), which function is unclear. Similarly, mTORC2 comprises mTOR and other five subunits: the scaffold protein RAPTOR-independent companion of TOR (RICTOR) and mammalian stress-activated protein kinase-interacting protein 1 (mSIN1), which could stabilize each other and maintain the stable structure of mTORC2; DEPTOR that is a negative regulator of mTORC2; mLST8, which is essential for the stability and activity of mTORC2; and protein observed with RICTOR (PROTOR), whose function is unclear [6]. For their difference in compositions, mTORC1 and mTORC2 have great differences in their sensitivities to rapamycin; in the upstream signals, they integrate; in the substrates, they regulate; and in the biological processes, they control [7].

As rapamycin could effectively inhibit the activity of mTORC1, the signal pathways and functions about mTORC1 have been well studied. As a serine/threonine kinase which could sense and respond to environmental cues, mTOR could respond to a series of environment signals, including glucose, amino acids, growth factors, and WNT signaling. The PI3K-AKT pathway is the main signaling pathway on its upstream, of which tuberous sclerosis complex (TSC) is one of the most important molecules regulating the activity of mTORC1. TSC, which is a Rheb-specific GAP, negatively regulates mTORC1 activity by inactivating Rheb activity [8]. mTORC1 regulates the activities of eukaryotic translation initiation factor 4E-binding protein 1 (4E-BP1), p70 ribosomal S6 kinase 1 (S6K1), protein phosphatase 2A (PP2A), transcription initiation factor IA (TIF-IA), sterol regulatory element-binding protein 1 (SREBP1), unc-51-like kinase 1 (ULK1), and autophagy-related gene 13 (ATG13) in its downstream [9–12]. Through these critical downstream molecules, mTORC1 plays important roles in lots of biological processes, including cell growth, proliferation, survival, autophagy, lipid synthesis, mitochondrial metabolism, and biogenesis.

mTORC1 plays positive roles in regulating cell growth and proliferation. It mainly promotes many biological processes of anabolism, such as biosynthesis of proteins, lipids, and lysosome. Meanwhile, it also suppresses biological processes of catabolism, for example, autophagy (Figure 1). Eukaryotic initiation factor 4E-binding protein 1 (4E-BP1) and p70 ribosomal S6 kinase 1 (S6K1) are two important downstream molecules of mTORC1, which are phosphorylated by mTORC1 and play critical roles in mTORC1-mediated protein synthesis. Phosphorylated 4E-BP1 could not bind to eIF4E, which enables eIF4E to promote cap-dependent translation. The stimulation of S6K1 activity by mTORC1 leads to the increase of mRNA biogenesis, cap-dependent translation and elongation, and the translation of ribosomal proteins. Meanwhile, the activation of mTORC1 has also been shown to promote ribosome biogenesis by stimulating the transcription of ribosomal RNA through a process involving the protein phosphatase 2A (PP2A) and the transcription initiation factor IA (TIF-IA) [9].

mTORC1 regulates cellular metabolism and biogenesis. The resting naïve T cells rely on the tricarboxylic acid (TCA) cycle and oxidative phosphorylation to generate ATP [13], while activated T cells primarily rely on aerobic glycolysis to maintain the energy requirement for proliferation and differentiation [14]. mTOR is extensively involved in various cell metabolisms in T cells. mTORC1 signaling has been reported to directly or indirectly regulate the transcription and expression of many key enzymes about the uptake of glucose and glycolysis in T cells through metabolism-associated transcription factors, such as HIF*α* and MYC [15, 16]. The pentose phosphate pathway (PPP) is an anabolic program employed in the process of T cell activation [17]. mTORC1 directly regulates the expression of key enzymes in PPP. Meanwhile, the inhibition of mTORC1 activity by rapamycin treatment greatly decreases the expression of these genes [18]. The resting naïve T cells seem to rely on fatty acid oxidation, and mTOR seems to be involved in fatty acid oxidation in other cells. It has been reported that at the same time of inhibition of mTORC1-dependent glycolysis by rapamycin, the rate of fatty acid oxidation increased [19]. Moreover, Brown et al. found that mTORC1 blocked by rapamycin inhibited the process of fatty acid and other lipid synthesis through a reduced expression of acetyl-coenzyme A carboxylase I and mitochondrial glycerol phosphate acyltransferasea. In addition, mTOR has also been reported to be involved in mitochondrial metabolism. Schieke et al. proved that rapamycin could decrease the mitochondrial membrane potential, and oxygen consumption and cellular ATP levels and profoundly alter the mitochondrial phosphoproteome by inhibiting the activity of mTORC1 in cells [20]. It has been observed that rapamycin inhibits the expression of many genes involved in oxidative metabolism, while enhanced mTORC1 activity by mutations increases the expressions of these genes. Bentzinger et al. has proved that conditional deletion of RAPTOR in the mouse skeletal muscle could reduce the expressions of genes associated with mitochondrial biogenesis [21]. The transcriptional activity of a nuclear cofactor PPAR*γ* coactivator 1 (PGC1-*α*), which plays a critical role in mitochondrial biogenesis and oxidative metabolism, also has been proved to be controlled by mTORC1 [22]. Besides protein synthesis, lipid synthesis, and mitochondrial biogenesis, mTORC1 also regulates the biogenesis of lysosomes. It has been reported that mTORC1 negatively regulates lysosome biogenesis through a basic helix-loop-helix leucine zipper transcription factor, transcription factor EB (TFEB) [23]. TFEB could be phosphorylated by mTORC1, which would prevent its nuclear entry and its activity to promote the expression of lysosomal genes [24, 25]. It has been proved that mTORC1 positively regulates the activity of two transcription factors sterol regulatory element-binding protein 1 (SREBP1) and peroxisome proliferator-activated receptor-*γ* (PPAR*γ*) [10, 26], both of which control the expression of gene encoding proteins involved in lipid and cholesterol homeostasis. When mTORC1 was blocked with rapamycin, the expression and transactivation activity of PPAR*γ* were reduced [26]. Thus, mTORC1 is widely involved in cell metabolism and biosynthesis.

Autophagy is a kind of catabolic process that recycles long-lived and faulty cellular components and promotes protein turnover. When the nutrient is limited in cells, the process of autophagy will work to degrade organelles and protein complexes, which could provide biological materials to sustain anabolic processes and energy production. mTORC1 inhibition increases autophagy and vice versa. However, Thoreen et al. found that mTORC1 controls the process of autophagy through an unknown mechanism that is essentially insensitive to the inhibition by rapamycin [27]. Meanwhile, Ganley et al. found that mTORC1 controls autophagy through the regulation of a protein complex composed of three subunits, including unc-51-like kinase 1 (ULK1), autophagy-related gene 13 (ATG13), and focal adhesion kinase family-interacting protein of 200 kDa (FIP200). They also showed that ATG13 and ULK1 were phosphorylated by the mTOR signaling pathway in a nutrient-starvation-regulated manner [11].

mTORC2 was initially considered rapamycin insensitive but proved to be inhibited by prolonged rapamycin treatment lately [28]. However, due to the absence of the effective mTORC2 inhibitor, relative little knowledge about mTORC2 biology was acquired until now compared to mTORC1. The upstream signals that lead to mTORC2 activation are not well characterized yet. Growth factors have been considered a signal for regulating the mTORC2 pathway [3]. mTORC2 is mainly involved in the regulation of phosphorylation and activation of AKT/PKB, protein kinase C, and serum- and glucocorticoid-induced protein kinase 1(SGK1) [7]. Various genetic approaches have been used to reveal the functions of genes, of course, mTORC2 is no exception. Studies have demonstrated that mTORC2 plays important roles in many biological processes, such as cell survival, metabolism, proliferation, and cytoskeleton organization [29]. mTORC2 regulates cell survival, metabolism, and proliferation through AKT. Two sites of AKT could be phosphorylated, including Ser308 and Ser473. Ser308 could be phosphorylated by phosphoinositide-dependent kinase 1 (PDK1), both of which are on the upstream of the mTORC1 signal pathway. Guertin et al. found that Ser473 was phosphorylated by mTORC2; it has been proved by subsequent experiments that deficiency of mTORC2 components specifically impedes the phosphorylation of AKT at Ser473 and some AKT substrates [30, 31]. Two transcription factors, forkhead box protein O1 (FoxO1) and FoxO3a, which control the expression of genes involved in stress resistance, metabolism, cell cycle arrest, and apoptosis, are negatively regulated by mTORC2 [32]. Recently, mTORC2 has been proved to regulate SGK1 [33]. SGK1 activity is totally abrogated when mTORC2 is inhibited. SGK1 and AKT phosphorylate FoxO1 and FoxO3a on common sites; so, the negative effects of mTORC2 on FoxO1 and FoxO3 are through the phosphorylation of SGK1 and AKT. Besides, mTORC2 could regulate cytoskeletal organization. Knocking down mTORC2 components affected actin polymerization and cell morphology through promoting phosphorylation of protein kinase Ca (PKC*α*) and paxillin [34, 35]. But the detailed mechanism of how mTORC2 regulates the process has not been determined.

## 3. mTOR in the Activation and Proliferation of T Cells

mTOR is one of the most important molecule that plays central roles in the regulation of metabolism, protein synthesis, energy balance, proliferation, survival, and various diseases [3, 29]. Activation and proliferation are accompanied by a series of biological processes which are all associated with the activity of mTOR, including protein synthesis and energy metabolism. Recently, it was revealed that mTOR plays critical roles in regulating the activation and proliferation of CD4^+^ T cells.

mTOR is an important signaling molecule for full T cell activation. T cell anergy would happen when cells are treated with mTOR-specific inhibitor rapamycin even in the presence of costimulation [36, 37]. Zheng et al. demonstrated that phosphorylation of S6 kinase 1 at Thr421/Ser424, a downstream kinase of mTORC1, was associated with full T cell activation [38]. Meanwhile, full T cell activation accompanied with an increased expression of transferrin receptor (CD71), which is mediated by the mTOR signaling pathway. On the contrary, anergic T cells showed markedly less S6 kinase 1 Thr421/Ser424 phosphorylation and CD71 expression [38]. Yang et al. found that RAPTOR-deficient T cells showed marked defects in TCR-induced CD71 expression, whereas RICTOR-deficient T cells exhibited no significant defects [39]. Meanwhile, the reversal of anergy is associated with the activation of mTOR, rather than the proliferation. mTOR, especially mTORC1, may play a central role in integrating environment signals that determine the outcome of Ag recognition, activation, or tolerance [38]. However, it seems that there is a discrepancy about the function of mTOR on T cell activation. mTOR deficiency in CD4^+^ T cells do not disturb and affect the TCR-induced signaling cascade and naïve T cell activation [5]. After stimulation with anti-CD3 mAb and irradiated APCs, the expressions of CD69 and CD25, which are two activation markers of CD4^+^ T cells, in mTOR-deficient CD4^+^ T cells, were upregulated appropriately as WT CD4^+^ T cells. While CD4^+^ T cells deficient of RICTOR, a critical adaptor protein of mTORC2 and determines the activity of mTORC2, showed comparable upregulation of activation markers CD69 and CD25 when stimulated with anti-CD3/CD28 [40]. Furthermore, mTOR deficiency and RICTOR deficiency in CD4^+^ T cells did not impact the production of IL-2 by T cells upon anti-CD3 stimulation [5, 41]. However, RAPTOR-deficient naive CD4^+^ T cells, which was lost in the mTORC1 activity, substantially reduced IL-2 production by T cells stimulated with anti-CD3/CD28 mAbs [39]. Beyond that, mTOR also impacts T cell activation through a metabolic pathway. T cell activation was accompanied by metabolic reprogramming, from fatty acid *β*-oxidation and pyruvate oxidation via the TCA cycle to the glycolytic, pentose-phosphate, and glutaminolytic pathways [15]. MYC has been proved as a pivotal regulator in the metabolic reprogramming during T cell activation and mTOR pathway cross-talks with MYC-dependent glutaminolysis. The phosphorylation and protein levels of downstream effectors in the mTOR pathway were reduced in MYC-deficient cells or under glutamine starvation condition [15]. HIF1*α* is a well-known transcription factor downstream of mTORC1 and responsible for the glycolytic response downstream of mTORC1 [18]. During T cell activation and proliferation processes, HIF1*α* was highly induced at both the transcription and protein levels. However, lack of HIF1*α* in T cells has no significant effects on glycolysis or T cell proliferation following immediate T cell activation [15]. Hence, it is necessary to further investigate the roles of the mTOR-regulated metabolic pathways in T cell activation.

Wiederrecht et al. found that mTOR promoted cell cycle progression [42]. T cells deficient of mTOR could not upregulate cyclin D3, which is critical for cell proliferation, when stimulated with anti-CD3 mAb and APCs *in vitro*. T cells deficient of mTOR proliferated less than CD4^+^ T cells of WT mice *in vitro* and *in vivo*, while mTOR deficiency in T cells did not abolish the proliferative capacity and increased cell death [5]. Ohtsubo et al. found that mTOR activity was needed for T cells to proliferate upon TCR/CD28-initiated stimuli or IL-2-dependent cell proliferation. Meanwhile, in these processes, mTOR was also need for optimal expression cyclin E, which is an important cell cycle regulator in the G1 phase and plays a critical role in the progress of G1-S phase transition [43]. Deletion of Rheb or RAPTOR in T cells, which abrogates mTORC1 activity, decreased the proliferation rate when T cells were stimulated with anti-CD3 and irradiated APCs or anti-CD3/CD28 or under Th1 and Th2 condition *in vitro* [39, 41]. These results suggested that mTORC1 might partially influence the proliferative activity of CD4^+^ T cells through cyclin protein. However, the effects of mTORC2 on the proliferation of CD4^+^ cells were inconsistent. There was a study that reported that CD4^+^ T cells deficient of RICTOR did not change the proliferative capacity and survival of T cells [40]. Meanwhile, Lee et al. also found that RICTOR deficiency did not impact the survival and apoptosis of T cells, while they found that IL-4-dependent proliferation of RICTOR-deficient CD4^+^ T cells was attenuated [44].

## 4. mTOR in the Differentiation of CD4^+^ T Cells

CD4^+^ T cells function mainly by releasing various kinds of cytokines that are involved in many immune responses to viral, bacterial, fungal, and parasitic infections and tumor immunity [45–49]. CD4^+^ T cells are also important for pathological processes of autoimmune diseases, such as systemic lupus erythematosus (SLE) [50, 51], autoimmune type 1 diabetes (T1D) [52, 53], multiple sclerosis (MS) [54, 55], rheumatoid arthritis (RA) [56–58], inflammatory bowel disease (IBD) [59, 60], and allergic responses or atopic diseases [61–63]. Naïve CD4^+^ T cells can differentiate into different subsets driven by three kinds of signals. Firstly, these cells need to be activated by the interaction of TCR and antigen presented by MHC II, which is not sufficient for a full activation of these cells. Further activation is determined by the engagement of costimulatory molecules between APCs and T cells [64]. T cell anergy will occur without costimulation signals [65]. Finally, the cytokine immune microenvironment will mainly determine the differentiation of distinctive T subtypes during naïve CD4^+^ T cells activation [66].

Since Robert Coffman and Timothy Mossman established “Th1-Th2” theory in the 1990s [67], other CD4^+^ T cell subsets have been found gradually and named mainly according to their cytokine profiles. Until now, a total of 7 kinds of Th subsets has been found, including Th1, Th2, Th9, Th17, Th22, follicular helper T cells (Tfh), and regulatory T cells (Treg) cells [46, 68]. Among these CD4^+^ T cell subsets, Th1, Th2, Th9, Th17, and Th22 cells mainly secrete distinct cytokines to mediate adaptive immunity to a variety of pathogens and regulate the pathogenesis of various diseases. Tfh cells are specialized to provide help to germinal center (GC) B cells and, consequently, mediate the development of long-lived humoral immunity [69]. While, Treg cells are a subpopulation of T cells that are immunosuppressive and generally suppress or downregulate the induction and proliferation of effector T cells to maintain immune tolerance to self-antigens, preventing autoimmune disease [70, 71].

**Figure 2:**
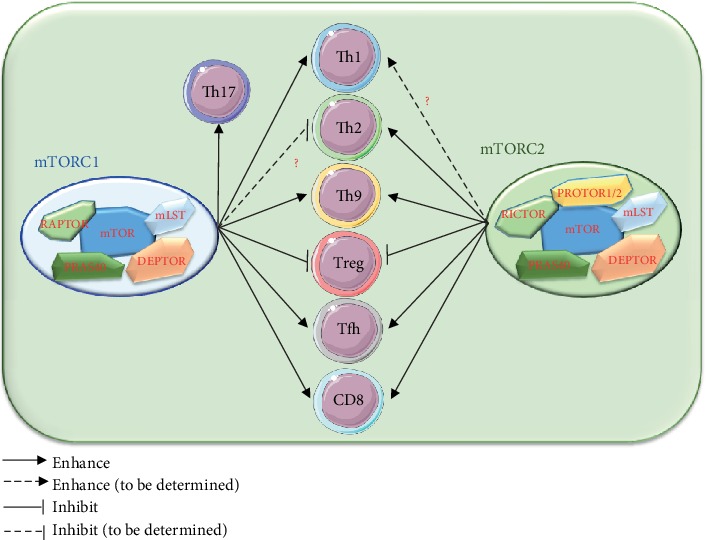
mTORC1 and mTORC2 differently regulate the differentiation of CD4^+^ Th cell subsets. Th1 and Th17 cell differentiation are positively regulated by mTORC1. Th2 cell differentiation is positively regulated by mTORC2. Both mTORC1 and mTORC2 positively regulate Th9, Tfh, and CD8 cell differentiation. Both mTORC1 and mTORC2 negatively regulate Treg cell differentiation.

Specific immune microenvironments determine the fate of naïve CD4^+^ T cells in the process of differentiation after they have been activated. After CD4^+^ T cells receive integrated cytokine signals from the microenvironment, a series of signal pathways and molecular events will be initiated, including a specific signal transducer and activator of transcription (STAT) transcription factor and other signaling molecules. Importantly lineage-specific transcription factors were then activated [46]. T-bet, GATA3, PU.1, IRF4, ROR*γ*t, AHR, Foxp3, and Bcl-6 are lineage-specific transcription factors of Th1, Th2, Th9, Th17, Th22, Treg, and Tfh cells, respectively [46, 68]. In addition to regulating cellular metabolism and other biological function by sensing and integrating environmental cues and signals [72], mTOR also significantly regulates the differentiation of CD4^+^ T cells, including Th1, Th2, Th9, Th17, Treg, and Tfh cells in response to a specific immune microenvironment [4] (Figure 2).

### 4.1. mTOR in Th1 Cells

Th1 cells are important for immunity to clear intracellular pathogens. mTOR has been found to play important roles in Th1 cell development both *in vitro* and *in vivo*. Under Th1 cell skewing conditions *in vitro*, CD4^+^ T cells deficient of *Frap1* (the gene that encodes mTOR protein) failed to differentiate into effector Th1 cells but did not change the expression levels of IL-12 receptor, which is important for Th1 cell differentiation [5]. Rheb is a crucial regulator of mTORC1 signaling, which could interact with mTORC1 to stimulate its activity [73]. Deletion of Rheb specifically in T cells almost completely abolished the activity of mTORC1 and preserved the activity of mTORC2, as indicated by the phosphorylation of S6K1 and phosphorylation of AKT at Ser473, respectively. Similar to T cells deficient of mTOR, T cells lacking Rheb failed to differentiate into Th1 cells under Th1 skewing conditions *in vitro* [41]. Since the proliferation activity of these T cells was slightly weaker than WT T cells, the inability of Rheb-deficient T cells to differentiate into Th1 cells was not caused by their lower rate of proliferation. Furthermore, in an antiviral Th1 response model induced by the *Vaccinia* virus *in vivo*, the CFSE-labeled WT and mTOR-deficient CD4^+^ T cells were adoptively transferred into host mice and immunized and showed that donor mTOR-deficient T cells could proliferate in response to viral infection, while they also failed to produce IFN-*γ* upon challenged with a high dose of antigen and APCs *in vitro* [5]. Similarly, in the antiviral Th1 response model induced by *Vaccinia* virus *in vivo*, compared to WT OT-II T cells, Rheb-deficient OT-II T cells produced significantly less IFN-*γ* while expressed substantial IL-4 [41]. Meanwhile, the inability to produce IFN-*γ* of these Rheb-deficient OT-II T cells *in vivo* was also not due to poor proliferation activity, for these cells failed to produce IFN-*γ* even those undergoing multiple rounds of cell proliferation. Meanwhile, Zeng et al. demonstrated that mTORC1 negatively controls Th1 cell differentiation in the process of LCMV-induced Th1 differentiation; they found that RAPTOR deficiency reduced T-bet^+^CXCR5^low^ Th1 cell number [74]. Therefore, mTORC1 is indispensable for Th1 cell differentiation both *in vitro* and *in vivo*. In contrast, the effects of mTORC2 in Th1 cell differentiation are contradictory. There is research reporting that T cells lacking mTORC2 activity were able to differentiate into Th1 under the appropriate induction conditions *in vitro*. In the antiviral Th1 response model induced by vaccinia virus *in vivo*, RICTOR-deficient T cells produced large amounts of IFN-*γ* as T cells of WT mice. Mice lacking RICTOR had similar clinical scores and pathology with WT mice in the EAE model, for the numbers of IFN-*γ*-producing Th1 cells in response to MOG peptide in mice specifically lacking RICTOR were equivalent to those in WT mice [41]. During the process of LCMV-induced Th1 differentiation *in vivo*, the number of Th1 cells even slightly elevated in RICTOR-deficient mice, in which the activity of mTORC2, is inactivated. However, Lee et al. found that conditional deletion of *RICTOR* in CD4^+^ T cells impaired Th1 cell differentiation *in vitro* [44]. Consistently, *RICTOR*-deficient mice also produced less frequency of IFN-*γ*-producing CD4^+^ T cells in lymphoid samples in an infectious challenge model. Meanwhile, these mice showed reduced IFN-*γ*-dependent IgG2a response when immunized with keyhole limpet haemocyanin (KLH) [44]. Hence, mTORC1 positively regulates Th1 cell differentiation, while the roles of mTORC2 in Th1 cell differentiation need to be further verified.

**Figure 3:**
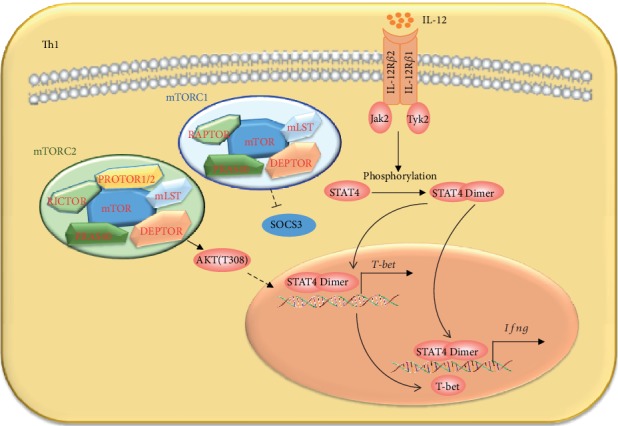
The regulation of mTOR on Th1 cell differentiation. Th1 cell differentiation is regulated mainly by mTORC1. mTORC1 positively regulates the expressions of *T-bet* and *Ifng* possibly through inhibiting the expression of SOCS3, which could inhibit the activation of STAT4. mTORC2 positively regulates Th1 cell differentiation through activating AKT, while the detailed mechanism has not been illuminated.

mTORC1 regulates Th1 cell differentiation through STAT4 and SOCS3, which then regulate T-bet expression [5, 41] (Figure 3). Rheb-deficient or mTOR-deficient T cells showed less phosphorylation of STAT4 in response to IL-12 and failed to fully upregulate lineage-specific transcription factor T-bet under Th1 induction conditions. Meanwhile, Rheb-deficient T cells expressed high levels of SOCS3 mRNA, and knockdown of SOCS3 mRNA resulted in higher T-bet expression and more IFN-*γ* production in Rheb-deficient T cells under Th1 induction conditions. Anyway, mTOR is essential for the upregulation of Th1 lineage-specific transcription factor T-bet, but the details of the molecular mechanisms need to be studied in the future [5]. mTORC2 regulates Th1 cell differentiation through AKT, for Th1 cell differentiation defect of *RICTOR*-deficient CD4^+^ T cells was rescued by the constitutively active mutant Myr-AKT and a mutant activated by phosphomimetic residues at T308. Moreover, active AKT also restored the T-bet expression in *RICTOR*-deficient CD4^+^ T cells under Th1 cell differentiation [44].

### 4.2. mTOR in Th2 Cells

Th2 cells are involved in allergic and atopic diseases and play important roles in host defense against parasites. mTOR has been reported to be indispensable for Th2 cell differentiation. Under Th2 cell skewing conditions *in vitro*, CD4^+^ T cell deficiency of *Frap1* failed to differentiate into effector Th2 cells, while it did not change the expression levels of IL-4 receptor, which is important for Th2 cells [5]. Compared to WT naïve T cells, RICTOR-deficient naïve T cells failed to differentiate into Th2 cells under Th2 cell induction conditions *in vitro* [41, 44]. Meanwhile, mice specifically lacking RICTOR had less IL-4-producing OVA-specific CD4^+^ T cells than WT mice in an OVA-elicited Th2 response. In contrast to the effect of mTORC2 on Th2 cell differentiation, it is inconsistent about the effects of mTORC1 on Th2 cell differentiation. Rheb-deficient T cells were able to differentiate into Th2 cells *in vitro*. Similarly, a very low dose of rapamycin, which only specially inhibit the activity of mTORC1, could specifically inhibit Th1 differentiation but not Th2 differentiation under Th2 induction conditions *in vitro* [41]. Consistent with the *in vitro* experiments, CD4^+^ T cells from mice deficient of Rheb produced identical and substantial IL-4 after immunization with OVA or infected with vaccinia virus compared to WT mice. Rheb-deficient OT-II T cells produced significantly less IFN-*γ* while producing substantial IL-4. Meanwhile, selective deletion of mTORC1 activity could lead to enhanced Th2 differentiation *in vivo*. Nearly 60% of MOG-immunized mice specifically lacking Rheb in T cells developed “nonclassical EAE,” a neurological disorder associated with MOG-specific Th2 cells and characterized by ataxia (not paralysis) and infiltration of the cerebellum with cells of the immune response [75, 76]. Compared to WT mice, mice lacking Rheb have more lymphocytes infiltrating the cerebellum and T cells isolated from these mice produced more Th2 cytokines after restimulation with MOG *in vitro* [41]. However, recent research demonstrated a central role of mTORC1 in Th2 cell responses both *in vitro* and *in vivo*. Yang et al. reported that lack of RAPTOR which also abolishes mTORC1 activity markedly decreased Th2 cell differentiation under Th2 conditions, with defective *IL-4* and *IL-13* mRNA expressions and IL-4 production. Moreover, RAPTOR-deficient mice showed attenuated lung inflammation and infiltration of leukocytes in the bronchioalveolar lavage (BAL) fluid in the OVA-induced allergic airway inflammation model. Hence, mTORC2 positively regulates Th2 cell differentiation both *in vitro* and *in vivo*, but mTORC1 seems to have different effects on Th2 cell differentiation, which needs more studies for verification.

**Figure 4:**
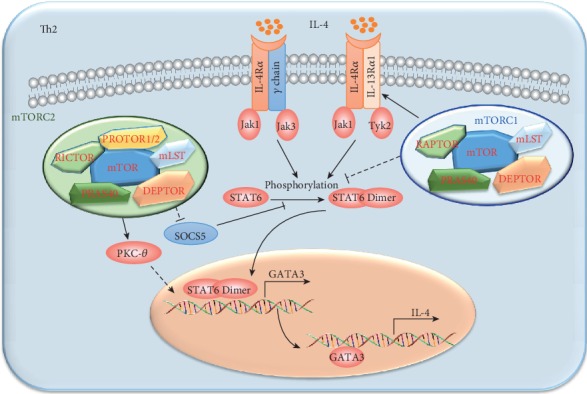
The regulation of mTORC1 and mTORC2 on Th2 cell differentiation. Th2 cell differentiation is regulated mainly by mTORC2. mTORC2 positively regulates the expressions of *GATA3* and *IL-4* through inhibiting the expression of SOCS5, which could inhibit the activation of STAT6. Meanwhile, mTORC2 positively regulates Th2 cell differentiation in a PKC-*θ*-dependent manner, while the detailed mechanism has not been illuminated. mTORC1 negatively regulates Th2 cell differentiation through modulating the activation of STAT6, while the detailed mechanism was not clarified.

mTORC2 regulates Th2 cell differentiation through STAT6, SOCS5, and PKC-*θ*, which then regulate the GATA3 expression [5, 41] and mTORC1 (Figure 4). The mTOR-deficient and RICTOR-deficient T cells showed diminished phosphorylation of STAT6 in response to IL-4 and failed to fully upregulate lineage-specific transcription factor GATA3 under Th2 induction conditions. Meanwhile, RICTOR-deficient T cells expressed high levels of SOCS5 mRNA, and knockdown of SOCS5 mRNA in RICTOR-deficient T cells resulted in higher GATA3 expression and a greater ability to produce IL-4. Lee et al. found that transduction of a constitutively active PKC-*θ* mutant reverted the GATA3 expression and the Th2 cell defect of *RICTOR*-deficient CD4^+^ T cells [44]. However, Rheb-deficient T cells showed an enhanced phosphorylated STAT6 level in response to IL-4, which also suggested that mTORC1 may slightly modulate Th2 cell differentiation. Meanwhile, RAPTOR-deficient T cells were unable to efficiently phosphorylate STAT6 and STAT5 and to increase GATA3 expression. Moreover, the reduced responsiveness to Th2-polarizing cytokines in RAPTOR-deficient T cells was due to defective induction of IL-4 and IL-2 receptors by defective mTORC1-dependent glucose metabolism. The precise roles and mechanisms for mTOR pathways in Th2 cell differentiation need to be clarified.

### 4.3. mTOR in Th17 Cells

Th17 cells are involved in host defense against extracellular bacteria, fungi, and other eukaryotic pathogens [68, 77]. mTOR is critical for Th17 cell differentiation both *in vitro* and *in vivo*. mTOR-deficient T cells failed to differentiate into Th17 cells. Further studies found that mTORC1 but not mTORC2 plays critical roles in Th17 cell differentiation. Compared to WT T cells, *Frap1*-deficient T cells, *RAPTOR*-deficient T cells, and *Rheb*-deficient T cells could not differentiate into Th17 cells under Th17 skewing condition *in vitro* [5, 41, 78], while *RICTOR*-deficient T cells could differentiate into Th17 cells in independent studies [5, 44]. Low doses of rapamycin strongly inhibited the differentiation of Th17 cells, even in the presence of IL-21 and IL-23 [78]. T cells isolated from Peyer's patches of mice which are specifically deficient of mTOR or Rheb in T cells showed less CD4^+^IL-17^+^ Th17 cells and produced less IL-17 after stimulation with PMA and ionomycin [5]. EAE is the most commonly used experimental model for the human inflammatory demyelinating disease and MS. Th1 and Th17 cells participate in promoting the pathology in EAE [79]. In the EAE model, mice specifically lacking Rheb in T cells had lower clinical scores and pathology, along with less infiltration of Th1 and Th17 cells in central nervous system. Gulen et al. found that SIGIRP, a negative regulator of IL-1 receptor and Toll-like receptor signaling, suppressed Th17 cell differentiation through suppressing the mTOR activity by inhibiting its phosphorylation. The mTOR-dependent inhibition of Th17 cell differentiation by SIGIRP was also found in the EAE model [80]. Through blockade of the mTORC1, rapamycin also has been proved to decrease Th17 cell differentiation in the EAE model and a murine CD4^+^ T cell transfer model of colitis [78]. These studies collectively demonstrated that mTORC1 plays the major role during Th17 cell differentiation both *in vitro* and *in vivo* [41].

**Figure 5:**
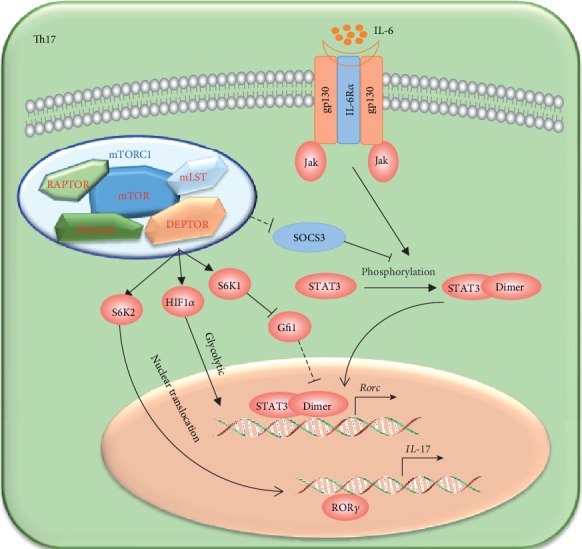
The regulation of mTOR on Th17 cell differentiation. Th17 cell differentiation is regulated mainly by mTORC1. mTORC1 positively regulates the *Rorc* expression and Th17 cell differentiation through the following pathways: (1) inhibiting the expression of SOCS3, which could inhibit the activation of STAT4; (2) regulating the expression of *Rorc* through the SGK1-Gfi1 signaling pathway; (3) promoting the nuclear translocation of ROR*γ* through S6K2; and (4) activating the HIF1*α*-dependent glycolytic pathway.

The regulatory roles of mTOR on Th17 cell differentiation through several distinct mechanisms were dependent on STAT3, transcription factor ROR*γ*t, and other molecules [5, 41, 78, 81] (Figure 5). IL-6 is important for Th17 differentiation, while T cells lacking mTOR did not impact IL-6 receptor expression. *mTOR*-deficient T cells and *Rheb*-deficient CD4^+^ T cells failed to increase STAT3 phosphorylation at tyrosine 705, which is indispensable for the gene expressions of IL-21 and ROR*γ*t during Th17 differentiation. Meanwhile, resting *Rheb*-deficient CD4^+^ T cells expressed a higher level of SOCS3 mRNA and protein than WT CD4^+^ T cells. After activation, these cells also highly expressed SOCS3. Hence, mTOR may regulate STAT3 and ROR*γ*t through SOCS3. Whether mTOR directly acts on STAT phosphorylation needs to be investigated. Meanwhile, mTOR-deficient T cells failed to upregulate the expression of IL-21, IL-23R, and ROR*γ*t, which are all important for the differentiation of Th17 cells. The PI3K-AKT-mTORC1 axis regulates Th17 cell differentiation through S6K1-mediated expression of Gig1, which negatively regulates Th17 cell differentiation by inhibiting ROR*γ*t activity. Meanwhile, the PI3K-AKT-mTORC1 axis could regulate nuclear translocation of ROR*γ* through S6K2 [78]. mTOR may also regulate Th17 cell differentiation through a metabolic pathway. HIF1*α*, which is induced by mTORC1, was selectively expressed in Th17 cells, and the HIF1*α*-dependent glycolytic pathway promoted Th17 cell differentiation. Rapamycin treatment inhibited HIF1*α* expression and HIF1*α*-mediated glycolytic activity and reduced the production of IL-17 under Th17 condition, which was similar to the effects of 2-deoxyglucose (2-DG), a prototypical inhibitor of the glycolytic pathway [16]. Meanwhile, HIF1*α*-deficient T cells did not impact the key molecules and transcription factors for Th17 cell differentiation, such as ROR*γ*t and STAT3, while significantly downregulating IL-23R expression under Th17-polarizing condition [16], which is essential for Th17 cell differentiation [82]. Hence, mTORC1 may regulate Th17 cell differentiation through the HIF1*α*-dependent glycolytic pathway.

### 4.4. mTOR in Treg Cells

**Figure 6:**
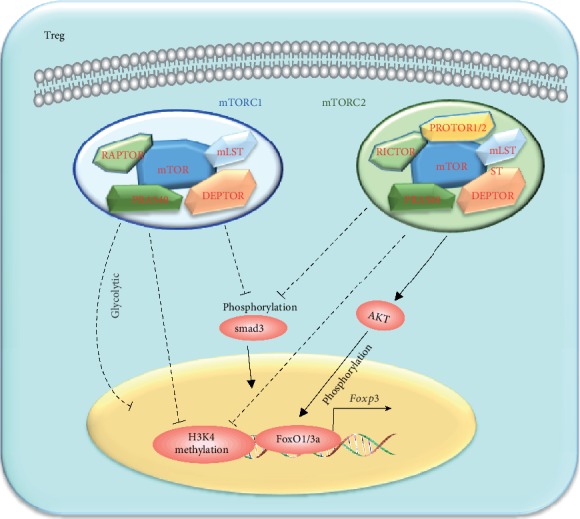
The regulation of mTOR on Treg cell differentiation. Treg cell differentiation is negatively regulated by mTORC1 and mTORC2. mTORC1 and mTORC2 inhibit Treg cell differentiation through inhibiting phosphorylation of smad3 or inhibiting H3K4 methylation near the Foxp3 TSS. Meanwhile, mTORC1 inhibits Treg cell differentiation by increasing glycolytic activity, but mTORC2 inhibits Treg cell differentiation through the ATK-FoxO1/3a pathway.

Treg cells play an essential role in regulating immune responses and in the induction and maintenance of peripheral tolerance via their suppressive function on effector CD4^+^ T cells and other immune cells [83]. Lots of researches have revealed that the mTOR signaling pathway is important for Treg cell differentiation, including natural Treg (nTreg) cells and induced Treg (iTreg) cells (Figure 6). It has been found that rapamycin selectively expanded the generation of murine naturally occurring CD4^+^CD25^+^Foxp3^+^ nTreg cells [84] and enhanced lasting induction of antigen-specific iTreg cells when accompanied with antigen administration [85]. Haxhinasto et al. reported that the AKT-mTOR axis could regulate de novo differentiation of Treg cells in the thymus and this signal axis did not affect the established Foxp3 expression in Treg cells [86]. Inhibition of PI3K/AKT/mTOR signaling pathway promoted Foxp3 expression and Treg-like gene expression profiles and microRNA expression profiles in CD4^+^ T cells, which suggests that PI3K/mTOR signaling controls not only Foxp3 and its direct targets, but also a wider Treg-like transcriptional program. In the contrary, constitutive PI3K/AKT/mTOR activity antagonized Foxp3 induction [87]. It is also reported that chemokine CCL3 regulated Foxp3 stability through mTORC2-PKB*α*/AKT1 serine 473 phosphorylation axis and played an important role in psoriasis [88]. Our results had revealed that TSC1 regulated thymic CD4^+^CD25^+^Foxp3^+^ nTreg cell development through a rapamycin resistant mechanism and an mTORC2-dependent signaling pathway [89]. All these findings indicate that mTOR signal regulates Treg cell differentiation and Foxp3 expression.

Naïve T cells will typically differentiate into Th1 cells under normal activating conditions, while mTOR-deficient T cells generated more functional CD4^+^CD25^+^Foxp3^+^ Treg cells under activation conditions (stimulated with anti-CD3 with APCs) with or without IL-2 *in vitro*. mTOR deficiency did not change the expressions of Foxp3 and glucocorticoid-induced tumor necrosis factor receptor (GITR) in Treg cells. Meanwhile, suppression assay showed that mTOR deficiency did not change the suppressive activity of Treg cells [5]. Similarly, Rheb-deficient or RICTOR-deficient T cells differentiate into more inducible Treg cells under iTreg skewing conditions (with TGF-*β*) *in vitro*. T cells lacking mTORC1 or mTORC2 activity did not impact the inhibitory functions of inducible Treg cells. Under activating conditions (without exogenous TGF-*β*), T cells differentiated into Foxp3^+^ Treg cells only when inhibiting both mTORC1 and mTORC2 activities [41]. Likewise, T cells in the absence of mTOR also differentiated into CD4^+^CD25^+^Foxp3^+^ Treg cells when activated by the strong Th1 cell-polarizing vaccinia virus infection *in vivo* and these Treg cells with potent suppressive capabilities [5]. All these studies suggest that mTOR signal negatively regulate Treg cell differentiation. Compared to WT T cells, mTOR-deficient T cells showed strong phosphorylation of smad3, which increased in the absence of TGF-*β* [5]. Thus, mTOR may increase sensibility of CD4^+^ T cells to TGF-*β* and activation of smad3. However, PI3K/mTOR inhibition induced by Foxp3 expression seems associated with H3K4 methylation near the Foxp3 transcription start site (TSS) and within the 5′ untranslated region (UTR) but not dependent on TGF-*β*-smad2/3 signaling, for neutralizing TGF-*β* antibodies, and the smad kinase inhibitor did not affect Foxp3 induction by PI3K/mTOR inhibitors [87]. As downstream protein molecules of the PI3K/AKT/mTOR axis, FoxO1 and FoxO3a could directly bind to the *Foxp3* promoter and link PI3K/AKT/mTOR to Foxp3 expression [90]. Harada et al. showed that inhibition of the PI3K/AKT/mTOR pathway restored Foxp3 induction [91]. The mTOR-dependent metabolic pathway is also important for Treg cell differentiation. Compared to T effector cells, Treg cells were less dependent on glycolytic activity and glycolytic enzymes. Contrary to Th17 cell differentiation, lack of HIF1*α* results in higher Foxp3^+^ Treg cell induction both *in vitro* and *in vivo* [16]. Meanwhile, either blocking mTORC1 by rapamycin or blocking glycolytic activity by 2-DG promoted Treg cell differentiation *in vitro* and *in vivo* [16]. However, the biochemical and molecular mechanisms of how mTOR regulates Treg cell differentiation has not been completely understood.

### 4.5. mTOR in Tfh Cells

Tfh cells are a subtype of Th cells, which were found in the periphery secondary lymphoid organs and constitutive expression CXCR5 [92]. They have been proved crucial for germinal center (GC) formation and play critical roles in the development of humoral adaptive immunity, which provide help to promote the affinity maturation and differentiation of B cells in GC follicles and produce high-affinity immunoglobulins [93]. As the specific transcription factor of Tfh cells, Bcl-6 masters the differentiation of Tfh cells [94].

mTOR, including mTORC1 and mTORC2, has been proved as an important regulator of Tfh cell differentiation. Ray et al. found that mTOR negatively regulates Tfh cell differentiation *in vivo*. Compared to Th1 cells, Tfh cells displayed a reduction in the activity of mTOR. mTOR could alter the balance of Tfh and Th1 cells *in vivo*. After CD4^+^ T cells, in which mTOR activity was silenced with an mTOR shRNA, are adoptively transferred to mice and infected with lymphocytic choriomeningitis virus (LCMV), the percentage of Tfh cells will increase while Th1 cells will significantly decrease. Furthermore, the authors found that IL-2 signaling via PI3K, AKT, and mTOR regulates the balance of Tfh and Th1 cell differentiation *in vivo* [95]. Zeng et al. reported that both mTORC1 and mTORC2 are important in Tfh cell differentiation and GC response [74]. mTOR signaling is indispensable for Tfh cell differentiation and GC formation in Peyer's patches (PPs). The frequencies of Tfh cells and B cells secreting IgA significantly decreased in PPs of CD4^cre^RAPTOR^fl/fl^ or CD4^cre^RICTOR^fl/fl^ mice under steady state. Meanwhile, deleting mTOC1 or mTORC2 in T cells significantly reduced Tfh cells in peripheral immune tissues when mice were infected with LCMV or immunized with OVA. Both mTORC1 and mTORC2 promote Tfh cell differentiation and GC formation intrinsically. mTORC1 and mTORC2 play crucial roles in linking ICOS signals to glucose metabolic and transcriptional regulation which promotes Tfh differentiation.

Furthermore, other studies indirectly proved that mTOR impacts Tfh cell differentiation through mTOR-associated molecules. PTEN is a molecule upstream of mTORC1 and negatively regulates the activity of mTORC1 [96]. The frequencies of GC B cells and Tfh cells significantly increased in PPs of mice deleting PTEN in CD4^+^ T cells, which suggest that PTEN negatively regulates Tfh cell differentiation and its function is consistent with mTORC1 [74]. PI3K, which is also an upstream molecule of mTORC1 and positively regulates its activity, has been proved a positive regulator of Tfh cell differentiation and cytokine production [97, 98]. These findings indirectly support a positive role of mTORC1 in Tfh cell differentiation. However, how mTOR regulates Tfh cell development has not been fully revealed. Zeng et al. found that mTORC2 promotes Tfh cell differentiation through FoxO1, for the reduction of FoxO1 activity in CD4^cre^RICTOR^fl/fl^ mice partially restored defective Tfh cells [74]. The detailed mechanism about how mTOR1 and mTORC2 regulate Tfh cell development and its transcription factor BCL-6 need to be further studied.

### 4.6. mTOR in Th9 Cells

Th9 cells are a Th cell subset which is defined by their high secretion of IL-9 in recent years. Th9 cells and its secreted IL-9 play both protective roles and pathological roles in inflammation and diseases. Th9 cells have been reported to be associated with lots of human diseases, such as atopic disease, IBD, EAE, and tumor immunity [99]. Many molecules and transcription factors are important in the regulation of Th9 cell development, including STAT6, STAT5, smad, BATF, PU.1, and IRF4. Among these molecules, PU.1 and IRF4 have been considered the relatively specific transcription factors for Th9 cells [100, 101].

**Figure 7:**
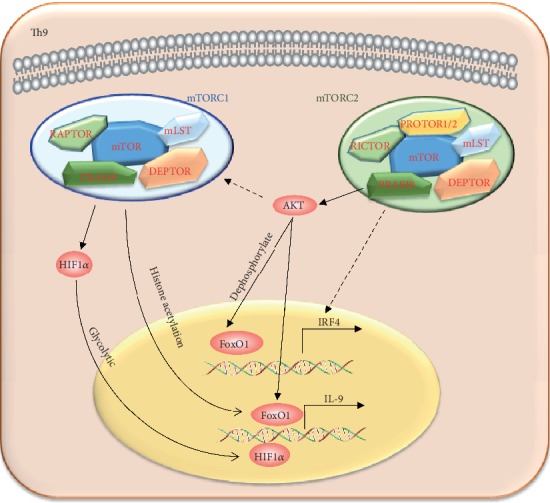
The regulation of mTOR on Th9 cell differentiation. Th9 cell differentiation is positively regulated by mTORC1 and mTORC2. mTORC1 positively regulates Th9 cell differentiation through the HIF1*α*-dependent glycolytic pathway and promotes the histone acetylation of *IL-9* promoter. mTORC2 positively regulates *IL-9* and Th9 cell differentiation mainly via the AKT-FoxO1 signaling pathway. mTORC2 also could regulate the expression of IRF4 through a FoxO1/FoxO3a-independetn pathway, while the detailed mechanism has not been illuminated.

mTOR plays critical roles in Th9 cell differentiation (Figure 7). Wang et al. reported that Th9 cell differentiation was associated with SIRT1-dependent glycolytic activity [102]. Meanwhile, they proved that HIF1*α* could directly regulate *IL-9* expression by binding to the *IL-9* promoter, and SIRT1-mTOR-HIF1*α* signaling-coupled glycolytic pathway positively regulated Th9 cell differentiation *in vitro* and in mouse tumor and allergic pulmonary inflammation models [102]. Bi et al. also found that the mTORC1 pathway could promote IL-9 expression by promoting histone acetylation at the *IL-9* promoter region, for rapamycin inhibited p300 abundance, histone acetylation at the *IL-9* promoter, and Th9 cell differentiation under Th9 skewing condition or plus with IL-7 [103].

Our recent studies demonstrate that mTORC2 plays important roles in Th9 cell development and Th9 cell-associated OVA-induced allergic airway inflammation [40]. Naïve *RICTOR*-deficient CD4^+^ T cells differentiated into significantly less Th9 cells and secreted less IL-9 in Th9 induction condition *in vitro*. Meanwhile, in a Th9 cell-associated OVA-induced allergic airway inflammation, mice specifically lacking RICTOR in T cells showed less Th9 cells and less severe allergic airway inflammation, which suggest that mTORC2 regulates Th9 cell development *in vivo*. FoxO1 and FoxO3a are two well-known important molecules downstream of mTORC2 [32]. Furthermore, we found that RICTOR deficiency impairs Th9 cell differentiation by reducing IRF4 expression rather than affecting the FoxO1/FoxO3a transcriptional activity [40], while the detailed mechanism about how mTORC2 regulates IRF4 remains to be addressed. However, Buttrick et al. proved that pharmacological inhibition of FoxO1 or genetic disruption of FoxO1 in CD4^+^ T cells reduced IL-9 expression *in vitro* and in an allergic airway inflammation model [104]. Meanwhile, they found that FoxO1 could directly bind to both *IL-9* and *IRF4* promoters and induced their transactivation [104]. Bi et al. also found that FoxO1 positively regulates IL-9 expression and Th9 differentiation when stimulated with IL-7 and plays important roles in the tumor model [103]. FoxO1 was dephosphorylated, translocated to the nucleus, bound to the *IL-9* promoter, and then promoted IL-9 expression when stimulated with IL-7 [103].

## 5. Summary

Besides the canonical Th1 and Th2 cells, more Th cell subsets have been proved to play vital roles in immunity and various inflammation and diseases in the past decades. The development and function have become the focus in immunology. As a central regulator of cell survival, cell proliferation, and cell metabolism, mTOR is critical for cell development and differentiation of the different subsets of T cells. Recent studies on the involvement of mTOR pathways in T cell biology will significantly enable us to better understand how these cells are regulated during differentiation and what roles these cells play in physiological and pathological conditions. Although we have achieved a basic understanding on how mTOR regulates T cell development, some questions still remain to be resolved, such as we did not know how mTORC1 regulates Th9 cell development. The detailed biochemical and molecular mechanisms of how mTOR regulates the lineage-specific transcription factors and genes have not been fully uncovered. With a detailed understanding of the roles and pathways of mTOR on T cell subset development and cytokine expression profiles, we may use mTOR inhibitors to treat T cell-associated inflammation and diseases more efficiently.

## Abbreviations

2-DG: 2-deoxyglucose
4E-BP1: 4E-binding protein 1
APCs: Antigen-presenting cells
ATG13: Autophagy-related gene 13
BAL: Bronchioalveolar lavage
CTLs: Cytotoxic T lymphocytes
DEPTOR: DEP-containing mTOR-interacting protein
FoxO1: Forkhead box protein O1
FIP200: Family-interacting protein of 200 kDa
GC: Germinal center
GITR: Glucocorticoid-induced tumor necrosis factor receptor
HP: Homeostatic proliferation
KLH: Keyhole limpet haemocyanin
mLST8: Mammalian lethal with Sec13 protein 8
mTOR: Mechanistic target of rapamycin
mTORC1: mTOR complex 1
mTORC2: mTOR complex 2
MS: Multiple sclerosis
mSIN1: Mammalian stress-activated protein kinase-interacting protein 1
IBD: Inflammatory bowel disease
LCMV: Lymphocytic choriomeningitis virus
RA: Rheumatoid arthritis
RAPTOR: Protein regulatory-associated protein of mTOR
RICTOR: RAPTOR-independent companion of TOR
TCA: Tricarboxylic acid
PGC1-*α*: The transcriptional activity of PPAR*γ* coactivator 1
PPP: Pentose phosphate pathway
PRAS40: Proline-rich AKT substrate 40 kDa
PI3KKs: Phosphatidylinositol 3-kinase-related kinases
PDK1: Phosphoinositide-dependent kinase 1
PKC*α*: Protein kinase C*α*
PROTOR: Protein observed with RICTOR
PP2A: Protein phosphatase 2A
PPAR*γ*: Peroxisome proliferator-activated receptor-*γ*
PPs: Peyer's patches
T1D: Autoimmune type 1 diabetes
TIF-IA: Transcription initiation factor IA
TFEB: Transcription factor EB
Tfh: Follicular helper T cells
TSC: Tuberous sclerosis complex
TSS: Transcription start site
S6K1: S6 kinase 1
SGK1: Serum- and glucocorticoid-induced protein kinase 1
SLE: Systemic lupus erythematosus
SREBP1: Sterol regulatory element-binding protein 1
STAT: Specific signal transducer and activator of transcription
ULK1: Unc-51-like kinase 1
UTR: Untranslated region.
